# Successful endoscopic hemostasis of arterial bleeding with concurrent technical troubleshooting during endoscopic sleeve gastroplasty using a single cinch

**DOI:** 10.1055/a-2643-8550

**Published:** 2025-07-25

**Authors:** Tung-Lung Wu, Hsu-Heng Yen, Yang-Yuan Chen

**Affiliations:** 1 36596 Division of Gastroenterology, Changhua Christian Hospital, Changhua, Taiwan; 2 34916 Department of Post-Baccalaureate Medicine, College of Medicine, National Chung Hsing University, Taichung, Taiwan; 3 36596 Artificial Intelligence Development Center, Changhua Christian Hospital, Changhua, Taiwan

As endoscopic sleeve gastroplasty (ESG) gains popularity as a minimally invasive bariatric technique, its overall complication rate remains low; nevertheless, intraprocedural bleeding is a well-recognized risk (reported incidence ≈1%) 1 . We report a case in which the suturing needle punctured a submucosal vessel during ESG, producing brisk arterial spurting and a rapidly expanding hematoma, compounded by needle misalignment that complicated further stitching ( Fig. 1 ). The hemorrhage appeared as pulsatile, bright-red flow, confirming an arterial source ( Video 1 ). Using the same OverStitch endoscopic suturing system already in place, the endoscopist deployed a single full-thickness stitch at the bleeding site and secured it with one cinch, achieving immediate hemostasis ( Fig. 2 ). No additional sutures, clips, or surgical intervention were required, and the procedure was completed uneventfully.

**Fig. 1:**
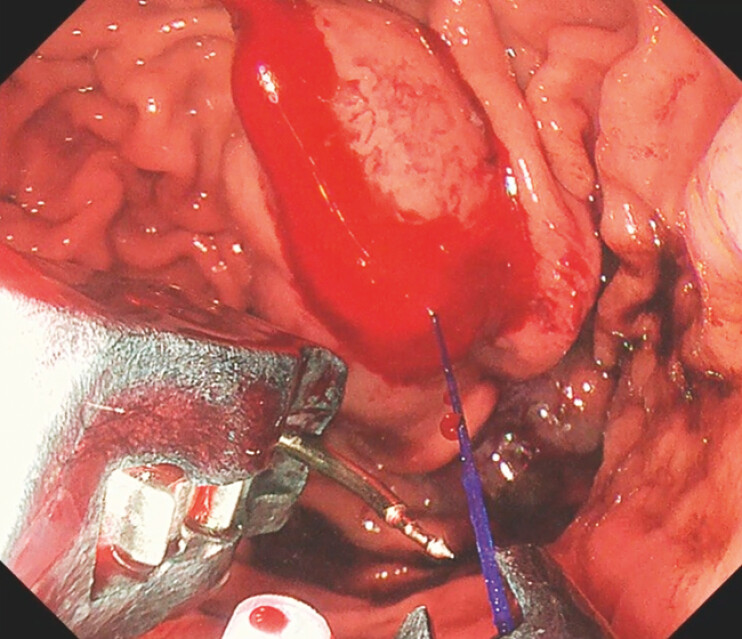
Arterial bleeding and hematoma formation following needle puncture, with visible needle misalignment.

**Fig. 2:**
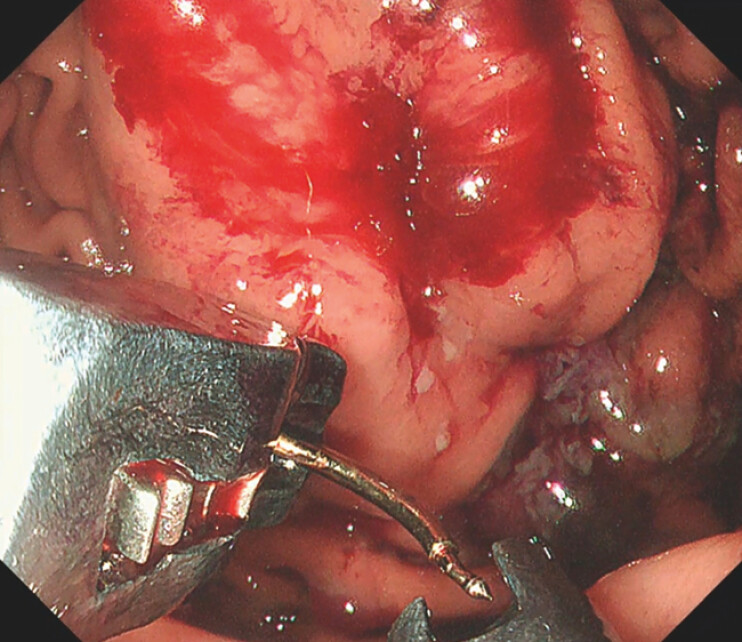
Bleeding clearly stopped after cinch placement, with no further expansion of the hematoma.

The video shows arterial bleeding during endoscopic sleeve gastroplasty (ESG), with hematoma formation following an accidental arterial puncture. A Cinch device was deployed to secure the suture, resulting in immediate hemostasis.Video 1

Choosing a single-stitch approach rather than escalating to more invasive measures was guided by the bleed’s focal location, its clearly visualized arterial spurting, and the suturing platform’s capacity for instantaneous tamponade. This case demonstrates that even substantial intraprocedural bleeding during ESG can be swiftly and effectively controlled with a single cinch, thereby avoiding surgical conversion and preserving the minimally invasive advantages of ESG, in which most adverse events can be managed non-operatively 2 .

Endoscopy_UCTN_Code_CPL_1AH_2AC

## Notes

Endoscopy E-Videos https://eref.thieme.de/e-videos
*E-Videos* is an open access online section of the journal *Endoscopy* , reporting on interesting cases and new techniques in gastroenterological endoscopy. All papers include a high-quality video and are published with a Creative Commons CC-BY license. Endoscopy E-Videos qualify for HINARI discounts and waivers and eligibility is automatically checked during the submission process. We grant 100% waivers to articles whose corresponding authors are based in Group A countries and 50% waivers to those who are based in Group B countries as classified by Research4Life (see: https://www.research4life.org/access/eligibility/ ). This section has its own submission website at https://mc.manuscriptcentral.com/e-videos .
